# Clinical trials on curcumin in relation to its bioavailability and effect on malignant diseases: critical analysis

**DOI:** 10.1007/s00210-023-02825-7

**Published:** 2023-11-15

**Authors:** Marten A. Khosravi, Roland Seifert

**Affiliations:** Institute of Pharmacology, Hannover Medical School, Carl-Neuberg-Straße 1, D-30625 Hannover, Germany

**Keywords:** Curcumin, Curcuminoids, Turmeric, Malignant diseases, Bioavailability, pubmed.gov, clinicaltrials.gov

## Abstract

Curcumin is an ingredient of the root *Curcuma longa*, which is responsible for the characteristic yellow color of curcuma. Curcumin is said to have the potential ability to fight malignant diseases and to have an anti-inflammatory effect. In addition, it is used as a dietary supplement. However, one problem with the use of curcumin is its extremely low bioavailability. The aim of this study is to systematically review and critically analyze clinical studies related to the pharmacokinetics (or bioavailability) and to the use of curcumin in the treatment of malignant diseases. The platforms clinicaltrials.gov and PubMed served as the database for the literature research. A total of 293 available studies on curcumin were filtered according to their focus (bioavailability, therapy of malignant diseases) and other criteria (study results, main substance, topic reference, existing disease/other research purpose, reference to malignant diseases). The studies were further analyzed regarding their outcome measures, their design (number of participants, randomization, placebo group, masking, ethical standards, sponsor, primary outcome measures, secondary outcome measures, study bias) and their findings. The analysis failed to convincingly demonstrate that curcumin has a significant, positive effect on the therapy of malignant diseases. Regarding the increase in bioavailability, positive results have been obtained, which are in proximity to the pharmaceutical industry. Independent studies could not achieve increased bioavailability of curcumin. The available reviews in the literature also do not provide convincing evidence for the efficacy of curcumin. Thus, at the time being, the use of curcumin in malignant diseases is not justified scientifically.

## Introduction

**Fig. 1:**
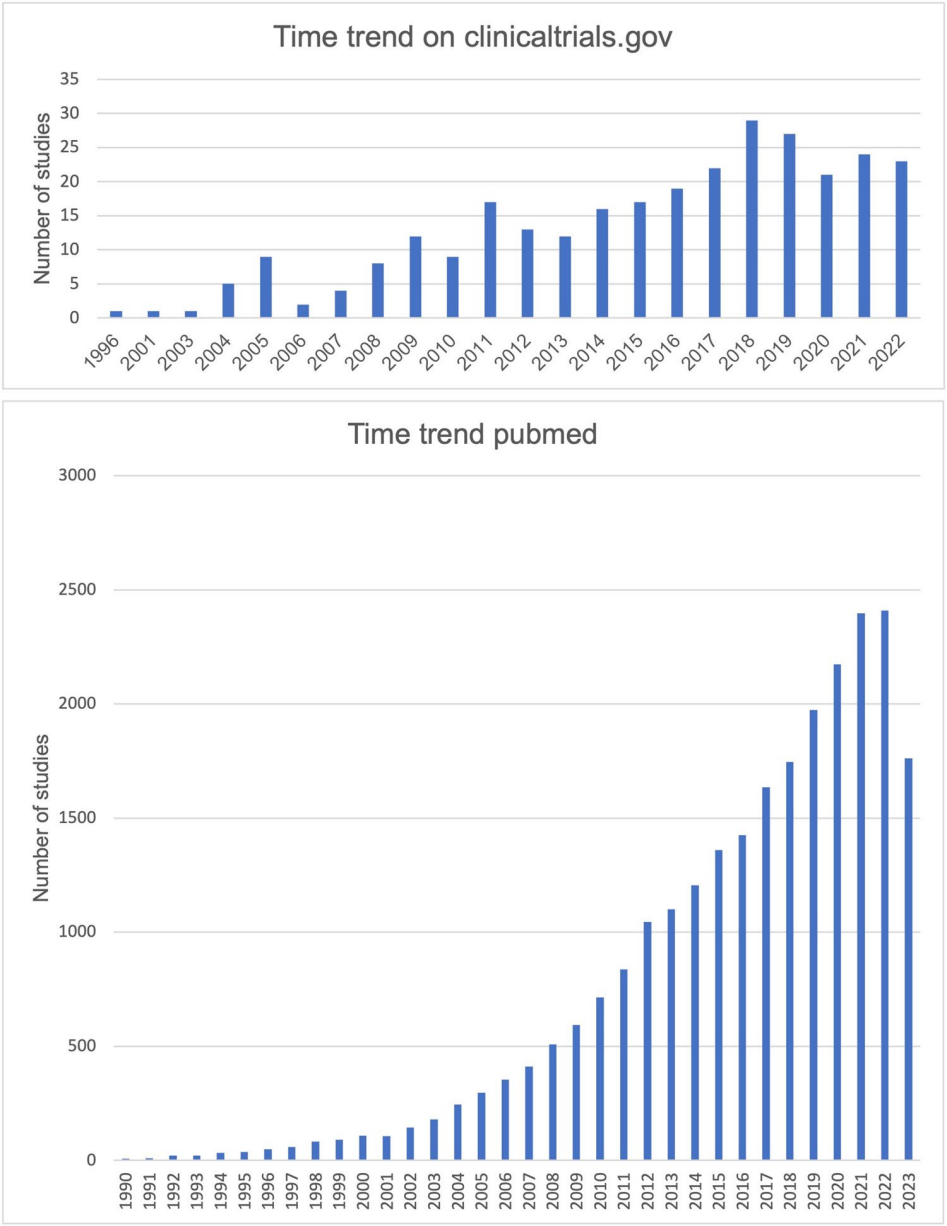

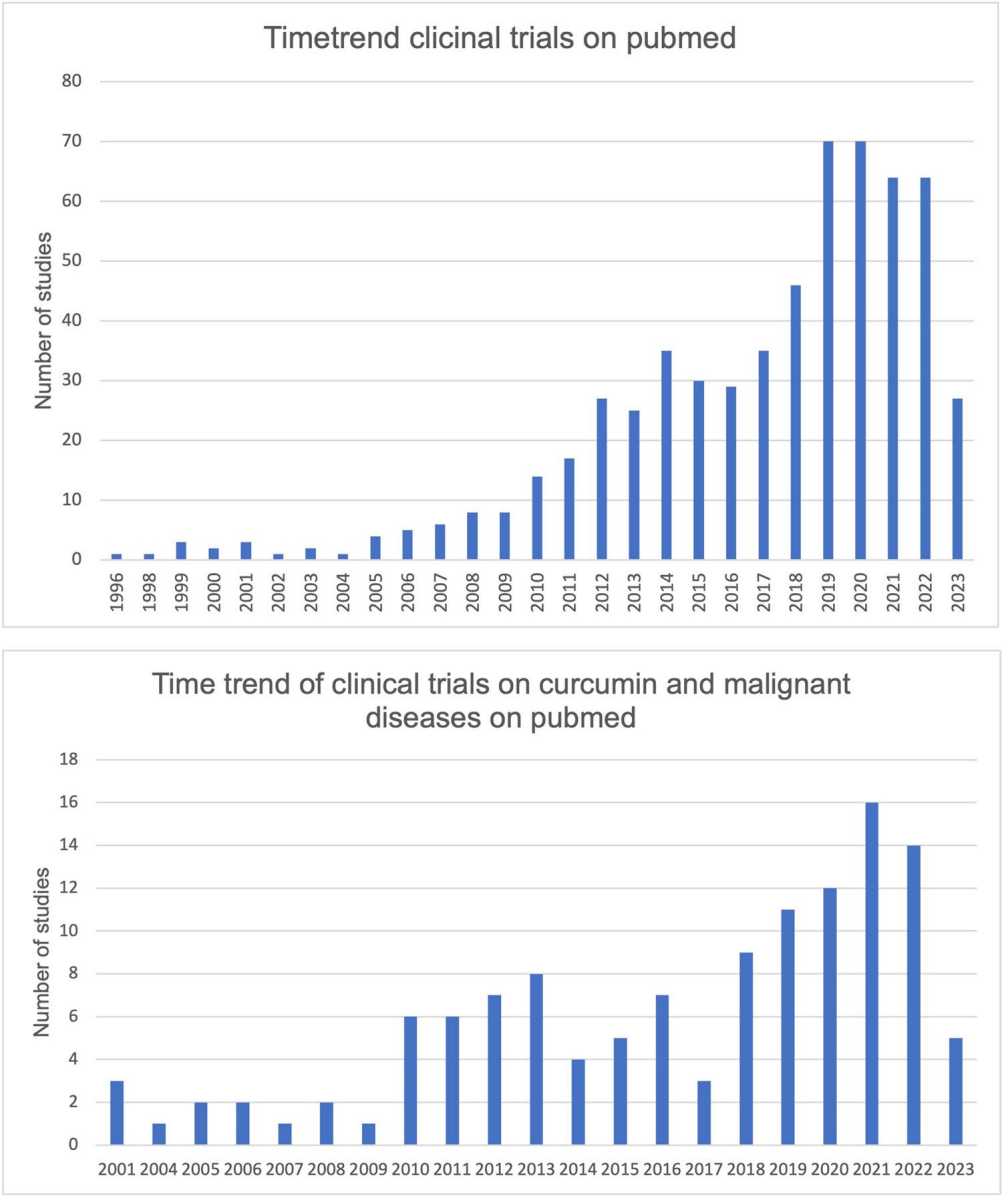
**a** The development over time of the clinical studies registered on curcumin on clinicaltrials.gov. **b** Timeline of studies on all scientific studies on curcumin on pubmed.gov (https://pubmed.ncbi.nlm.nih.gov/?term=curcumin, accessed 22 Septmeber 2023). **c** Time trend of clinical trials on curcumin on pubmed (https://pubmed.ncbi.nlm.nih.gov/?term=curcumin&filter=pubt.clinicaltrial, accessed 22 September 2023). **d** Time trend of clinical trials on the effect of curcumin on malignant diseases on pubmed (https://pubmed.ncbi.nlm.nih.gov/?term=curcumin+cancer&filter=pubt.clinicaltrial, accessed 22 September 2023)

**Fig. 2:**
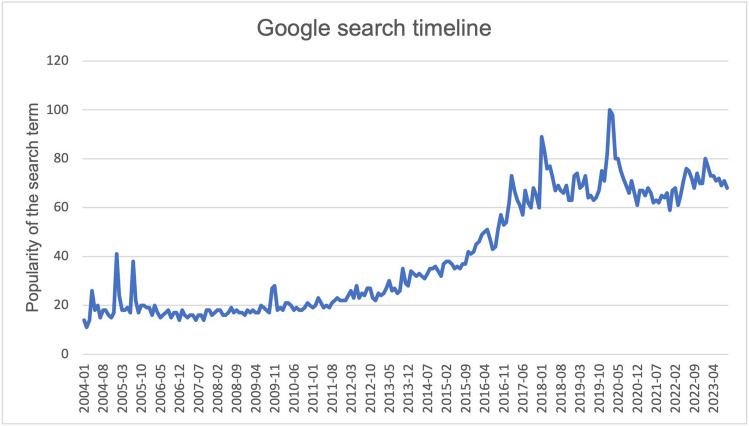
Development over time of Google searches for curcumin (https://trends.google.com/trends/explore?date=all&q=%2Fm%2F01vlkr&hl=de, accessed 22 September 2023)

Malignant diseases continue to pose significant challenges to the healthcare system (https://health.ec.europa.eu/system/files/2022-02/eu_cancer-plan_en_0.pdf, accessed 25 September 2023). The search for innovative therapeutic options is, therefore, highly relevant and for some years has included the naturally occurring active ingredient curcumin from the root curcuma. A true hype has arisen around curcumin and its potential effect on malignant diseases, which also had an impact on its research. Figure 1 shows the increasing time trend of studies on curcumin on clinicaltrials.gov and pubmed.gov. However, interest in curcumin is not only growing in the scientific community; the general public is also trying to find out more about the substance and its potential effects, as shown by Google searches (Fig. 2). The root *Curcuma longa*, which is also widely used as a spice in Asia, contains various curcuminoids (Fança-Berthon et al. 2021). Curcumin is one of these curcuminoids and is metabolized in the body in two metabolic phases (Liu et al. 2016). In phase 1 metabolism, NADPH-dependent reduction occurs, and in phase 2 metabolism, the metabolites are made more water-soluble by conjugation with glucuronides and sulfates and prepared for excretion (Liu et al. 2016). After oral ingestion, only a small portion of curcumin is absorbed. The absorbed portion is metabolized at a pH of > 7 within 20 min (Liu et al. 2016). After absorption, phase 1 and phase 2 metabolism occur mainly in the liver and plasma, followed by excretion in the urine (Liu et al. 2016). Therefore, the problem of curcumin bioavailability can be attributed to its low absorption, rapid metabolism, short half-life, and low tissue distribution (Anand et al. 2007). The low bioavailability of curcumin is a limiting factor regarding the pharmacological effect of the drug. In this critical analysis, we analyze studies on the efficacy of curcumin against malignant diseases as well as studies on ways to increase bioavailability and thereby pharmacological efficacy. Our data are placed into the context of three review articles in the literature.

## Methods

**Fig. 3:**
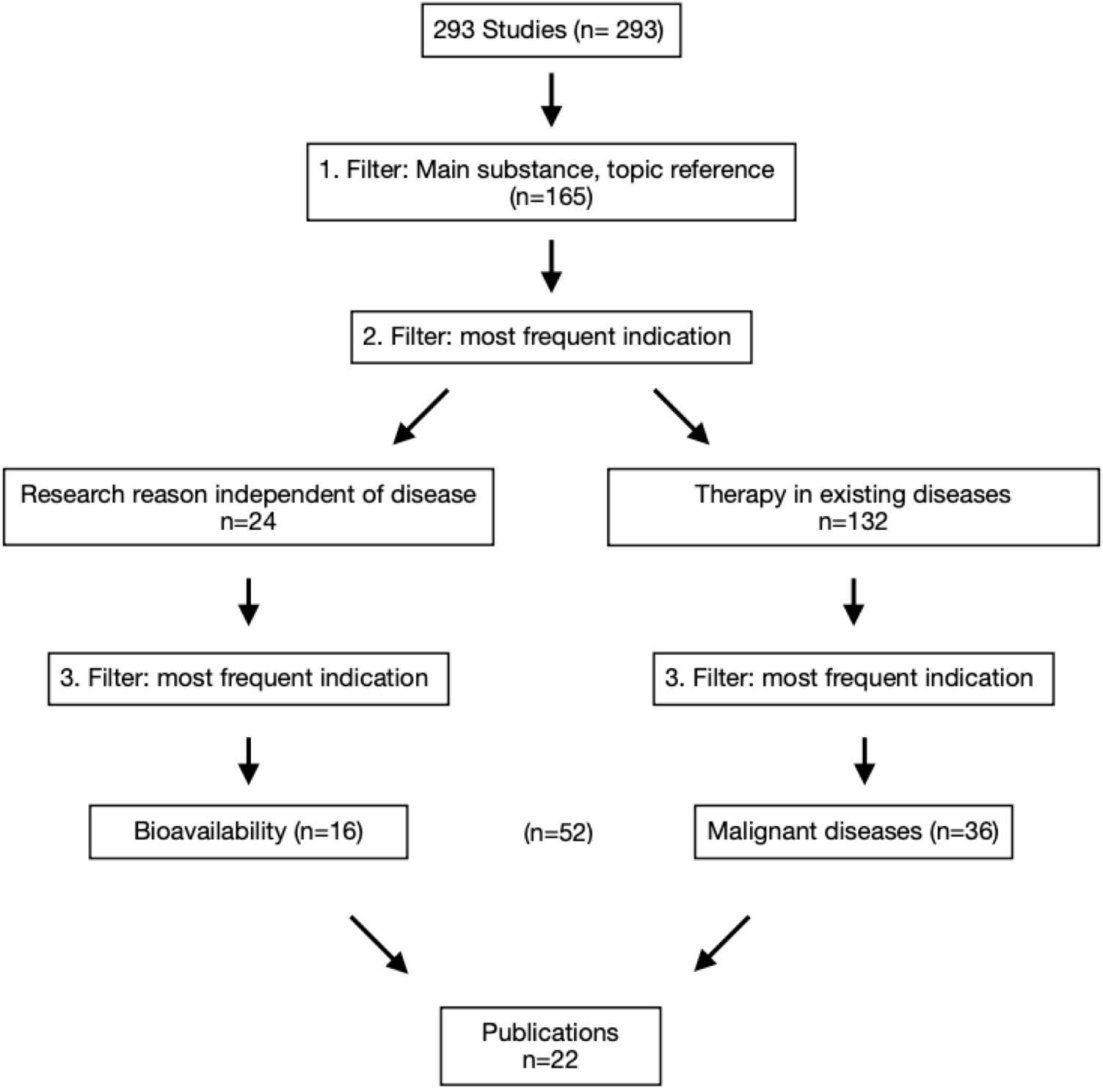
The filtering process of all analyzed studies

The studies analyzed were limited to clinical trials, listed on the clinicaltrials.gov website. For the keywords curcumin and curcuma, 293 studies were found. From these 293 clinical trials, all studies relevant to this work were filtered out in 3 rounds of filtering (Fig. 3). All studies were included in Excel tables and were compared and analyzed.

In the first filtration, the studies were examined for the following parameters: main substance, topic reference, study results on clinicaltrials.gov or pubmed.gov, use of curcumin in existing diseases, reference to therapy of malignant diseases, research in healthy subjects. Using these parameters, the studies were narrowed down to 165 trials. These clinical trials were now divided into two main groups: the use of curcumin in existing diseases and the use in healthy subjects. From each of these main groups, the most common indications were determined. The analysis was then limited to the two most frequent indications (bioavailability and curcumin in relation to malignant diseases). The detailed analysis was performed on 52 studies of the same indications. In order to further differentiate studies related to the effect of curcumin in malignant diseases, the studies were divided into two further categories. **Active treatment** describes studies that deal with active parameters of the treatment of malignant diseases (for example, inhibition of tumor growth). **Treatment of concomitant complaints** describes studies that deal with the effect of curcumin on the side effects of malignant diseases or the therapy of malignant diseases (for example, fatigue, radiation dermatitis).

**Fig. 4:**
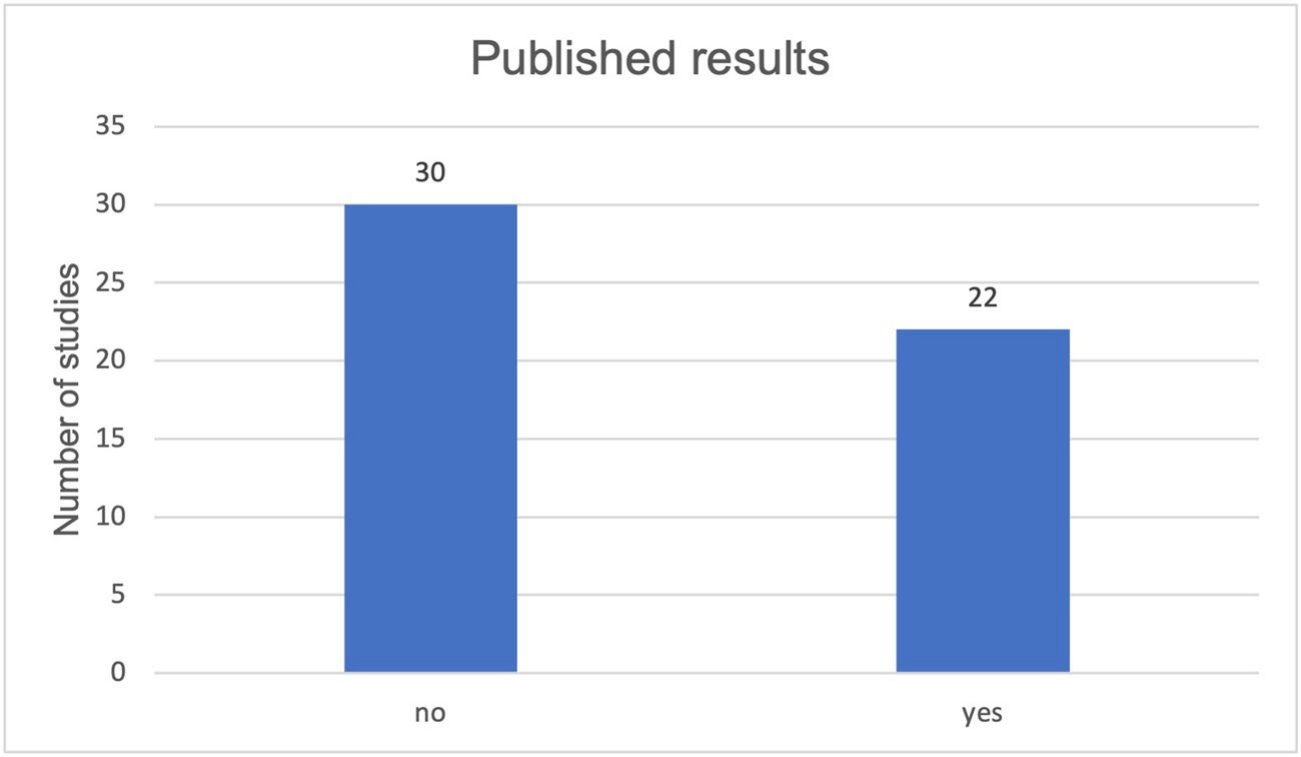
Proportion of studies with published results out of all studies analyzed

The 52 studies were divided into groups, based on their publication status. Both groups were analyzed and compared with the following parameters: placebo group, location, gender, average age of subjects, condition, enrollment, actual number of participants, patient dropouts, randomization, way of administration, combined dose/day, formulation of curcumin, duration of application, primary outcome measure timeframe, reference to malignant diseases (active treatment, treatment of concomitant complaints). The Student *T*-test was used to compare the data obtained from these two groups (studies with and without published results) and to verify significant differences. Just 22 out of the 52 selected studies revealed published results (Fig. 4). Subsequently, all published results were evaluated and analyzed regarding their outcome measures. Each study was checked for study bias during the method critique.

## Results

### Comparison between studies with and without published results

The applied *T*-test found significant differences in the comparative parameter “combined dose/day” between studies with and without publications. Studies with published results used on average twice the dose of curcumin (5.015 g versus 2.54 g). Phase I clinical trials showed that curcumin is safe for humans even at high doses (12 g/day), but still has low bioavailability (Anand et al. 2007). So far, no relation between increased dosage and improved bioavailability of curcumin has been proven.

**Fig. 5:**
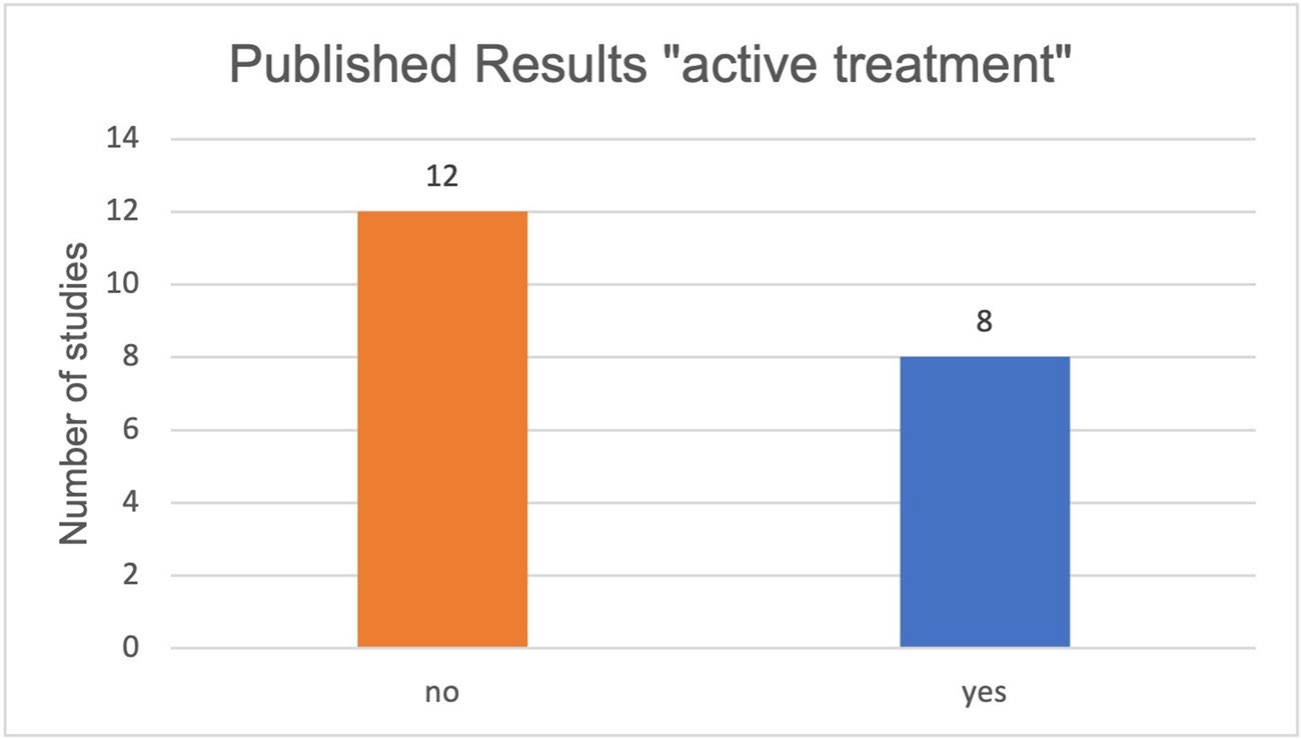
Ratio of studies with and without publications in the active treatment category

**Fig. 6:**
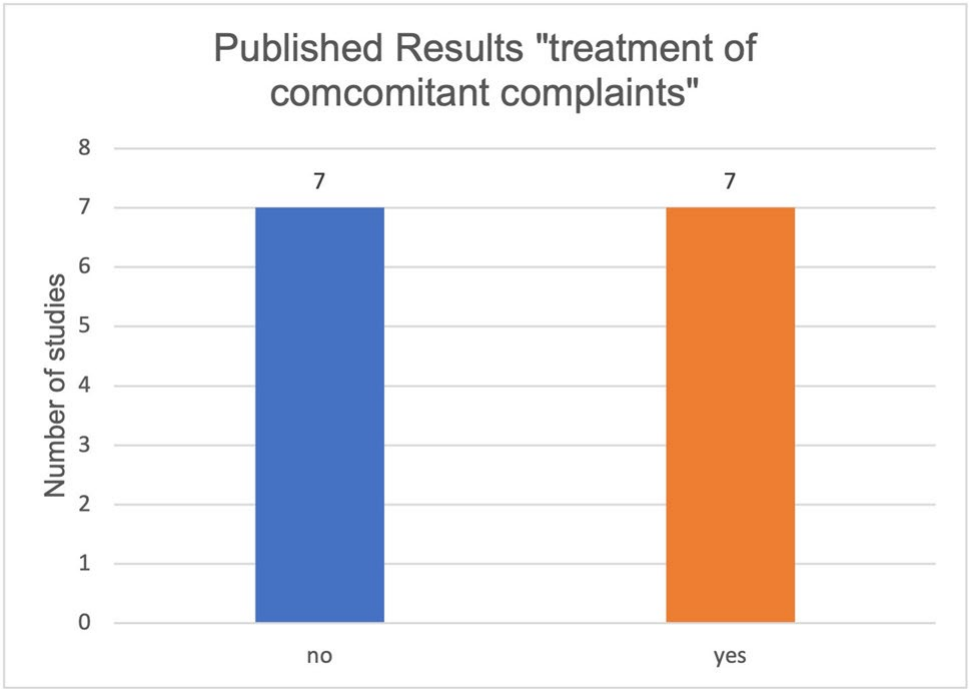
Ratio of studies with and without publications in the category Treatment of concomitant complaints

**Fig. 7:**
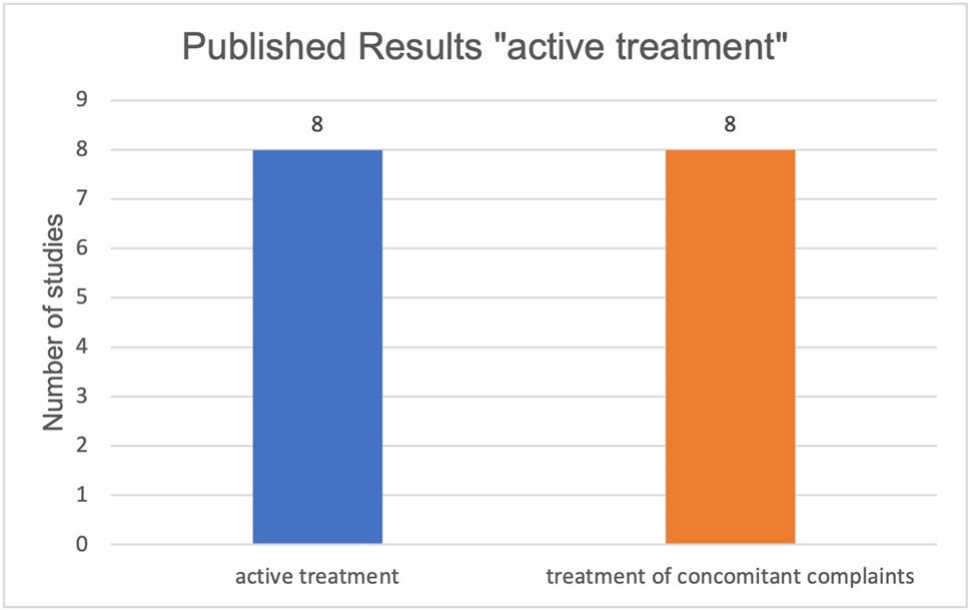
Distribution of all published studies in relation to curcumin and malignant diseases

**Fig. 8:**
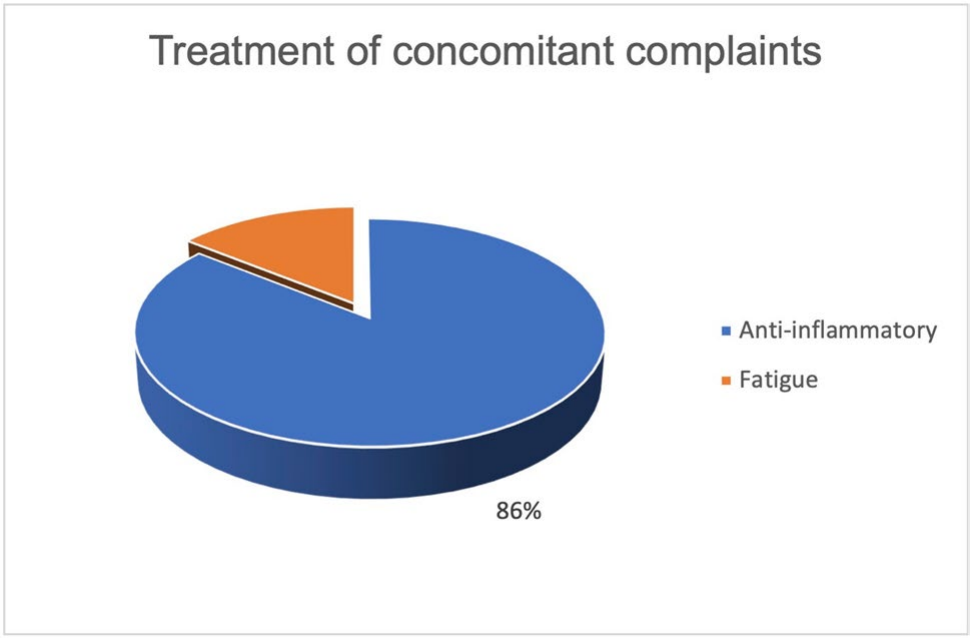
Outcome measures of publications in the category of treatment of concomitant complaints

Furthermore, the parameters of active treatment and treatment of concomitant complaints in relation to curcumin and malignant diseases were also studied in relation to published and unpublished results. Sixty percent of the studies on active treatment of malignant diseases by curcumin did not have published results (Fig. 5). Fifty percent of the studies on the treatment of side effects of the therapy of malignant diseases by curcumin have published results (Fig. 6). Regarding all published studies on curcumin and malignant diseases, 50% of the studies investigate the “active treatment.” Fifty percent of the studies investigate the “treatment of concomitant complaints” (Fig. 7). The anti-inflammatory effect of curcumin was investigated as the primary outcome measure in 86% of the published studies concerning the “treatment of concomitant complaints” (Fig. 8). Accordingly, half of the existing publications about curcumin and malignant diseases are not about the actual effect of curcumin on the same malignant diseases, but about the control of side effects of the therapy. The side effects examine to 86% the anti-inflammatory effect of curcumin. The anti-inflammatory effect has been examined in 2020 in a metanalysis of 32 clinical trials (Ferguson et al. 2021).

### Use of curcumin in malignant diseases

**Fig. 9:**
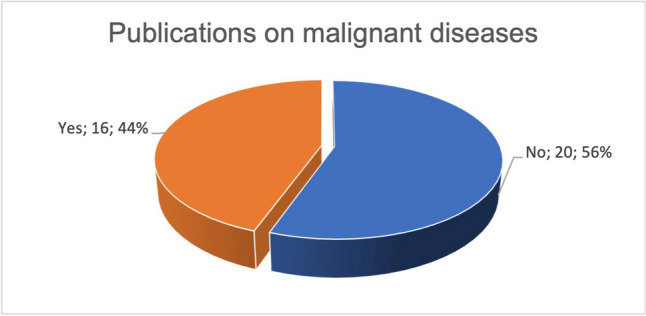
Proportion of publications in relation to all studies available on the subject of curcumin and malignant diseases

Of a total of 36 studies on malignant diseases, 16 studies were published (Fig. 9).

**Fig. 10:**
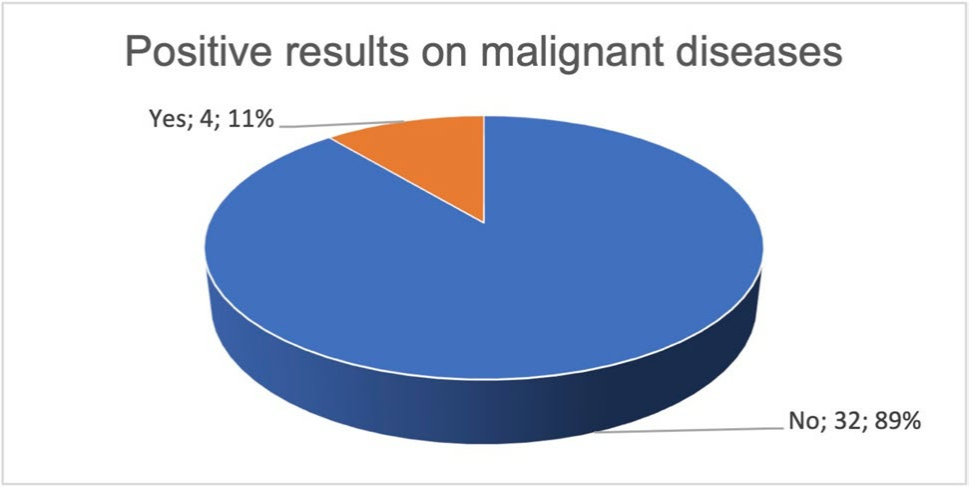
Proportion of studies with proved, primary outcome measures regarding all available studies, on the effect of curcumin on malignant diseases

**Fig. 11:**
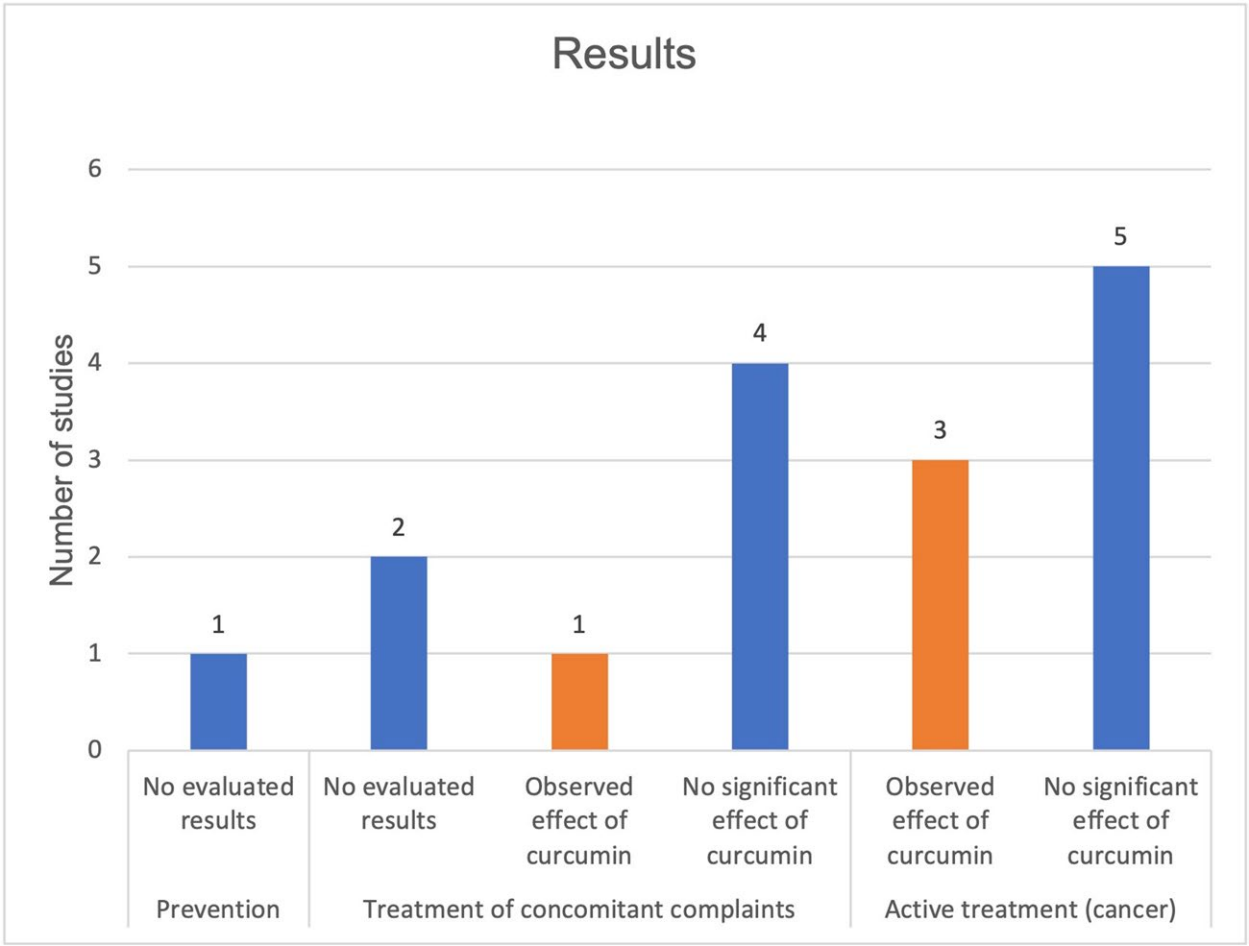
Proportion of studies with proven, primary endpoints out of all available studies on the effect of curcumin on malignant diseases, regarding the categories active treatment and treatment of concomitant complaints

Four studies achieved the primary outcome measures and thereby an effect of curcumin (Fig. 10). Figure 11 shows the published studies that achieved their primary outcome measures in relation to the predefined categories “active treatment” and “treatment of concomitant complaints.” Out of a total of 7 publications, one publication in the category “treatment of concomitant complaints” achieved the primary outcome measures. Out of 8 publications in the category “active treatment,” three studies achieved the primary outcome measures. A total of four publications with positive primary outcome measures, in relation to the effect of curcumin on malignant diseases, were analyzed. Table 1 presents the method critique and indicates possible study bias of the studies with positive research results. Two out of four publications on the effect of curcumin and malignant diseases are burdened by study bias. The detailed results of the studies are listed in combination with the method critique in the discussion.

**Table 1:** Method critique of the studies that successfully proved their primary outcome measures

NCT number	Sample selection	Number of participants	Randomization	Placebo group	Masking	Ethical standarts	Sponsor	Primary outcome measure	Bias/confounding	References
NCT00094445	Appropriate medical diagnosis with stable general condition	25	No	No	None (open label)	Observed	Public facility	Participant Survival	None randomized, no placebo group, primary outcome measure	Dhillon et al. (2008)
NCT04208334	Appropriate medical diagnosis with stable general condition	18	Yes	Yes	Quadruple	Observed	Public facility	Muscle mass	None	Thambamroong et al. (2022)
NCT03072992	Appropriate medical diagnosis with stable general condition	133	Yes	Yes	Triple	Observed	Public facility	Response Rate	None	Saghatelyan et al. (2020)
NCT00969085	Appropriate medical diagnosis with stable general condition	8	No	No	None (open label)	Observed	Public facility	Response Rate	In vitro	Zhang et al. (2010)
NCT01925287	Healthy	23	No	No	Single	Observed	Relevant company	Plasma concentration	Selection, none randomized, no placebo group	Schiborr et al. (2014)
NCT01403545	Healthy	50	Yes	Yes	Triple	Observed	Relevant company	Tolerability	Sponsor	Storka et al. (2015)
NCT03621865	Healthy	30	Yes	No	None (open label)	Observed	Relevant company	Plasma concentration	Sponsor, no placebo group, masking	Fança-Berthon et al. (2021)
NCT04382014	Healthy	31	Yes	No	Quadruple	Observed	Relevant company	Plasma concentration	Sponsor, no placebo group	Aguilera et al. (2022)
NCT04028739	Healthy	24	Yes	No	None (open label)	Observed	Relevant company	Plasma concentration	Sponsor, no placebo group	Chung et al. (2021)

### Bioavailability

**Fig. 12:**
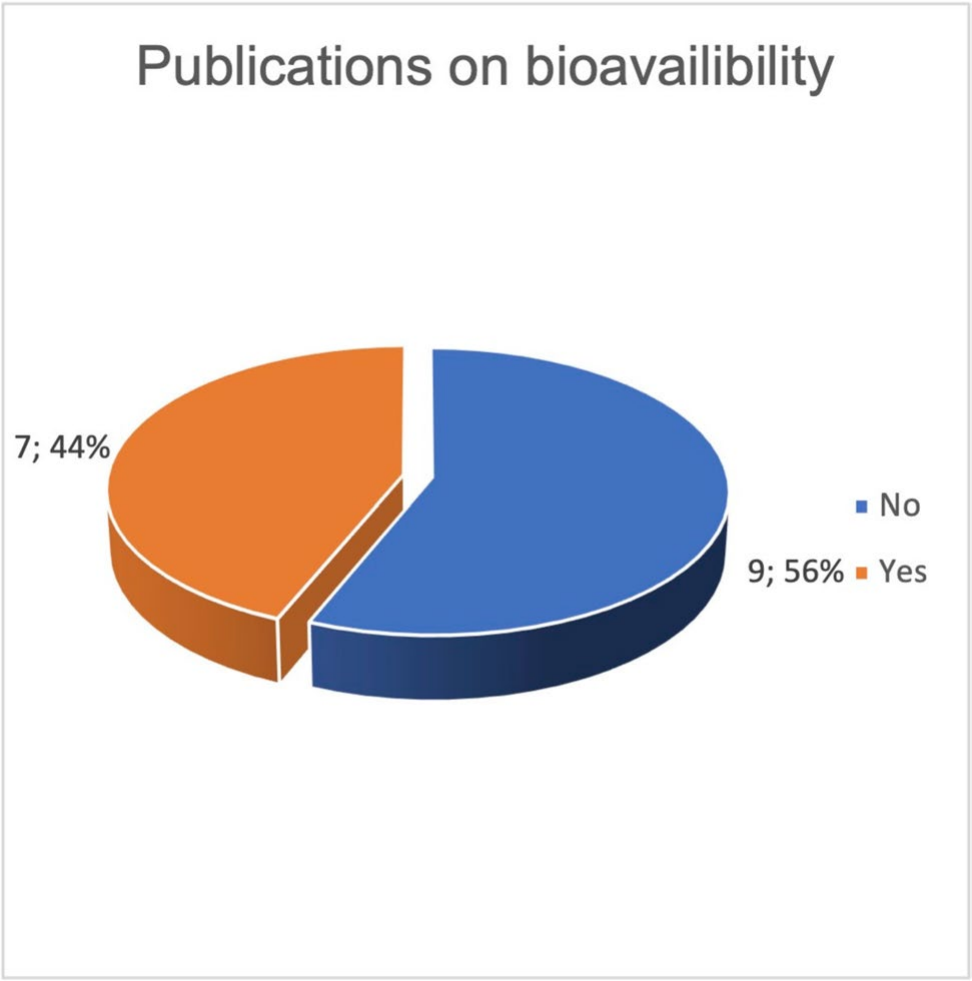
Proportion of studies published on the topic of curcumin bioavailability

**Fig. 13:**
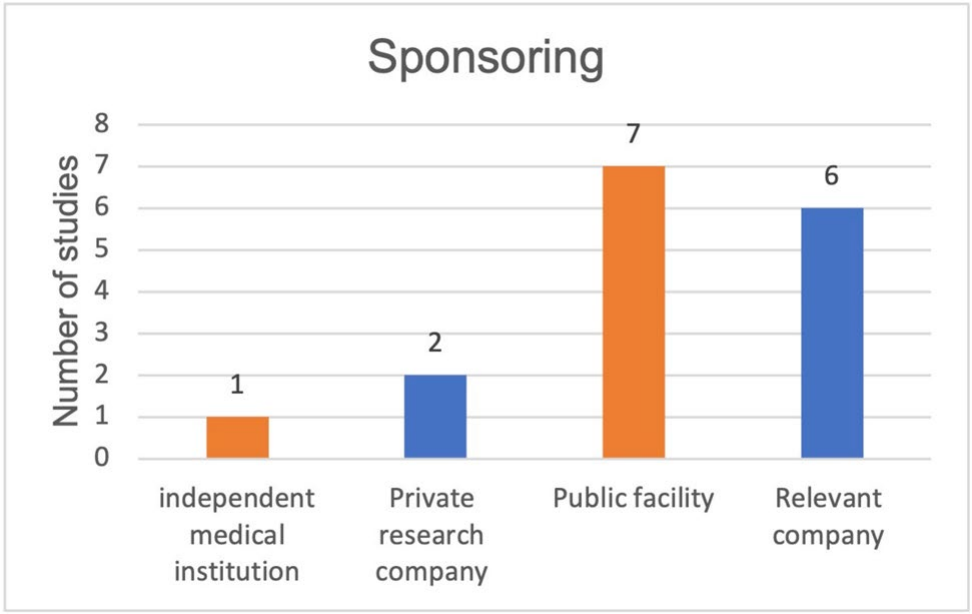
Proportion of industry-sponsored studies in relation to all available bioavailability studies

Out of a total of 16 studies on clinicaltrials.gov, seven studies were published (Fig. 12). Six of these published studies were able to successfully prove their primary outcome measures. Similar to studies on malignant diseases, study biases were frequently found in studies on the bioavailability of curcumin. Table 1 shows the industry sponsoring of the studies. Four out of 7 studies on bioavailability were funded by pharmaceutical companies. Every study that successfully proved a higher bioavailability of curcumin was founded by an industrial company (Fig. 13). Out of a total of 16 studies, 15 investigated the bioavailability of curcumin after oral consumption. Only one study investigated the effects of intravenous administration of curcumin. The detailed results of the studies are listed in combination with the method critique in the discussion.

## Discussion

Table 1 shows the method critique of all published studies with successfully proven primary outcome measures. The very right column lists any study bias. Study biases were found in 11 out of 13 studies after the methodological review. Table 2 shows all published studies with their corresponding results.

**Table 2:** Precise results of the studies, that successfully proved their primary outcome measure

Study	Pimary outcome measure	Sponsoring	Reference	Result	Precise results	References
NCT00094445	6-month participant survival	Public facility	Active treatment	Observed effect of curcumin	Decreased activity of transcription factors such as NF-κB, COX-2, and STAT3	Dhillon et al. (2008)
NCT04208334	Muscle mass	Public facility	Treatment of concomitant complaints	Observed effect of curcumin	Significant increase in muscle mass. The reduction in absolute lymphocyte count was significantly lower	Thambamroong et al. (2022)
NCT03072992	Objective response rate	Public facility	Active treatment	Observed effect of curcumin	ORR was significantly higher. The combination of curcumin and paclitaxel resulted in a significantly higher response rate	Saghatelyan et al. (2020)
NCT00969085	Response Rate using Physician's Global Assessment (PGA) based on Severity-Weighted Assessment Tool (SWAT)	Public facility	Active treatment	Observed effect of curcumin	Curcumin induces apoptosis in Cutaneous T-Cell Lymphoma (CTCL) cell lines.	Zhang et al. (2010)
NCT01925287	Plasma concentration	Relevant company	Bioavailability	Enhanced bioavailability of curcumin	Curcumin micelles and micronized curcumin led to significantly higher concentrations of curcumin, DMC, and BDMC in the plasma compared to native curcumin.	Schiborr et al. (2014)
NCT01403545	Safety and tolerability of increasing doses	Relevant company	Bioavailability	Enhanced bioavailability of curcumin	Short-term intravenous dosing of liposomal curcumin appears to be safe up to a dose of 120 mg/m^2^. Changes in red blood cell morphology may represent a dose limiting sign of toxicity.	Storka et al. (2015)
NCT03621865	Plasma concentration	Relevant company	Bioavailability	Enhanced bioavailability of curcumin	The micellar curcumin formulation (NOV) and the colloidal suspension Turmipure Gold (TPG) led to significantly higher concentrations of curcumin and its metabolites in the plasma compared to the standard turmeric extract (STE). Phytosome formulation (PHYT) showed lower absorption.	Fança-Berthon et al. (2021)
NCT04382014	Plasma concentration	Relevant company	Bioavailability	Enhanced bioavailability of curcumin	One hour after ingestion of a single dose, the combination of curcuminoids with MAG-OM3 resulted in a more rapid increase of curcuminoids in blood plasma compared to other formulations	Aguilera et al. (2022)
NCT04028739	Plasma concentration	Relevant company	Bioavailability	Enhanced bioavailability of curcumin	Theracurmin formulations (CR-033P and CR-031P)had significantly higher Cmax and AUClast values compared to the non-formulated curcumin powder	Chung et al. (2021)

### Malignant diseases

#### Curcumin on the expression of COX-2 and NF-κB

A clinical study on the effect of curcumin on patients with pancreatic cancer investigated the influence of curcumin on the expression of COX-2 and NF-κB (Table 2, NCT00094445). COX-2 expression levels decreased significantly after oral administration of 8 g curcumin/day (*p* < 0.03, Student’s *t* test) (Dhillon et al. 2008). A downregulation of NF-κB was also observed but did not reach statistical relevance (Dhillon et al. 2008). Despite the downregulation of both factors, no clinical response was observed in many patients, which was attributed in the study to the measurement in PBMC, which does not reflect what is occurring in the tumor itself (Dhillon et al. 2008). This study was not randomized and did not include a placebo group (Table 1, NCT00094445). In addition, another study analyzed in our work found no significant change in COX-2 expression with oral administration of 2 g curcumin/day (Tuyaerts et al. 2019). However, this study conducted 11 years later was only performed on 7 participants and did not have a placebo group or randomization. Both studies are affected by study bias and do not allow a clear statement on the effect of curcumin on COX-2 expression due to the different results. COX-2 was investigated regarding its overexpression in tumor cells, which contributes to tumor growth. The idea was to inhibit tumor growth by inhibiting COX-2. The role of COX-2 inhibitors in tumor therapy has been investigated in clinical trials and reported as promising in a review (Hashemi Goradel et al. 2019). Successfully demonstrating the downregulation of COX-2 would not create a new treatment method, but at most an alternative to the already existing COX-2 inhibitors such as celecoxib and etoricoxib.

#### Cutaneous T-cell lymphoma (CTCL)

An in vitro study investigated the antitumoral effect of curcumin on CTCL cell lines (Table 2, NCT00969085). It observed that curcumin could induce apoptosis in CTCL cells (Zhang et al. 2010). Furthermore, an increased apoptosis rate was found in patients with an increased percentage of circulating tumor T-cells compared to healthy subjects (Zhang et al. 2010). The study was conducted as an in vitro study. Especially with a compound like curcumin, which is characterized and limited by its low bioavailability, an in vitro study can only provide directional results (Table 1, NCT00969085). The complex pharmacokinetics cannot be disregarded; therefore, the results of the study cannot be directly transferred to the human organism.

#### Curcumin in combination with paclitaxel in patients with breast cancer

A study investigated the efficacy of curcumin in combination with the chemotherapeutic drug paclitaxel in patients with metastatic breast cancer (Table 2, NCT03072992). The subjects were divided into a paclitaxel and curcumin combination group and a group with paclitaxel as monotherapy (Saghatelyan et al. 2020). A significantly higher objective response rate (ORR) was observed in patients receiving curcumin in combination with paclitaxel (Saghatelyan et al. 2020). The ORR in the curcumin group was 50.7%, while the paclitaxel monotherapy group was 33.3% (*p*<0.05) (Saghatelyan et al. 2020). Treatment continued for 12 weeks and included a subsequent observation period of 4 weeks (Saghatelyan et al. 2020). Three months after the end of treatment, a re-analysis was performed on the subjects and the observed benefits of the treatment group with curcumin paclitaxel combination remained (Saghatelyan et al. 2020). A slightly increased PFS was observed in the curcumin group compared to the placebo group. However, the difference in PFS did not reach statistical significance (*p* = 0.3495) (Saghatelyan et al. 2020). No study bias was detected (Table 1, NCT03072992).

#### Increase in muscle mass in patients with anorexia-cachexia syndrome

Patients with locally advanced or advanced head and neck cancer were treated with 4 g curcumin (oral administration) over a period of 8 weeks. The primary outcome measure was muscle mass (Table 2, NCT04208334). Patients with head and neck cancer in particular lose a lot of skeletal muscle in anorexia-cachexia syndrome due to increased energy consumption and systemic inflammation (Thambamroong et al. 2022). Curcumin was supposed to counteract this through its anti-inflammatory characteristics (Thambamroong et al. 2022). The study observed an average gain in muscle mass of 0.46 kg muscle mass in the patients treated with curcumin, after the interval of 8 weeks (Thambamroong et al. 2022). Patients receiving placebo lost an average of 1.05 kg of muscle mass (Thambamroong et al. 2022). A significant difference was found between the placebo group and the subjects receiving curcumin (Thambamroong et al. 2022). An important factor here was the inhibition of the NF-κB signaling pathway, which is involved in the degradation of the skeletal muscles (Thambamroong et al. 2022). Inhibition of the NF-κB pathway has also been observed in patients with pancreatic cancer, as discussed earlier (Dhillon et al. 2008). No study bias was detected (Table 1, NCT04208334).

### Bioavailability

#### Micelles

Two of the studies analyzed investigated a possible increased bioavailability of curcumin due to a micelle compound (Table 2, NCT01925287, NCT03621865). It was observed that the maximum plasma concentration and the corresponding AUC were significantly higher after administration of curcumin micelles and micronized curcumin than after the administration of native curcumin (Schiborr et al. 2014). One of the studies observed 24 times higher bioavailability in a 24-h interval with a specific curcumin micelle formulation (Turmipure GOLD™) compared to curcuma standard extract (Fança-Berthon et al. 2021). In addition, a dose of 300 mg of the micellar curcumin combination was found to have a higher plasma concentration than a dose of 1500 mg of curcuma standard extract (Fança-Berthon et al. 2021). It is important to consider that the results of both studies were affected by industry sponsoring (Table 1, NCT01925287, NCT03621865). This study bias could lead to a reporting of the studies in favor of the contributing or funding company.

#### Intravenous, liposomal curcumin

Liposomal curcumin was studied intravenously at doses of 10–400 mg/m^2^ to determine its safety and bioavailability (Storka et al. 2015). Liposomal curcumin was infused into healthy volunteers at 2-h intervals. An increase in plasma concentrations of curcumin (Cmax 42–2575 ng/ml) and its metabolite tetra hydro curcumin (THC, Cmax 41–265 ng/ml) was observed (Storka et al. 2015) (Table 2, NCT01403545). After completion of the infusion, plasma concentrations decreased to undetectable levels within 6–60 min (Storka et al. 2015). In addition, an increase in the MCV of erythrocytes was observed from a dose of 120mg/m^2^ and higher, although the effect was reversible in all subjects (Storka et al. 2015); the change in erythrocyte morphology limits intravenous administration of liposomal curcumin above a dose of 120mg/m^2^ (Storka et al. 2015). This study was funded by a relevant pharmaceutical company and is therefore affected by industry sponsoring (Table 1, NCT01403545).

#### Curcuminoids in combination with omega-3 fatty acids

Four hundred milligrams of curcuminoids in powder form, 400 mg of curcuminoids with rice oil, and 400 mg of curcuminoids with omega-3 fatty acids mixed with monoglycerides (MAG-OM3) were compared in terms of plasma concentrations of curcuminoids (Aguilera et al. 2022). No difference was found between the 3 formulations in terms of maximum plasma concentration; all formulations reached their maximum plasma concentration after 4 h (Aguilera et al. 2022). After comparing the plasma concentrations of the 3 formulations 1 h after ingestion, a significantly higher concentration of curcumin and total curcuminoids was observed in the subjects of the MAG-OM3 group (Aguilera et al. 2022). The plasma concentration increased more quickly, although it could not exceed the maximum plasma concentrations of the other formulations (Aguilera et al. 2022) (Table 2, NCT04382014). This study is affected by industry sponsoring (Table 1, NCT04382014).

**Fig. 14:**
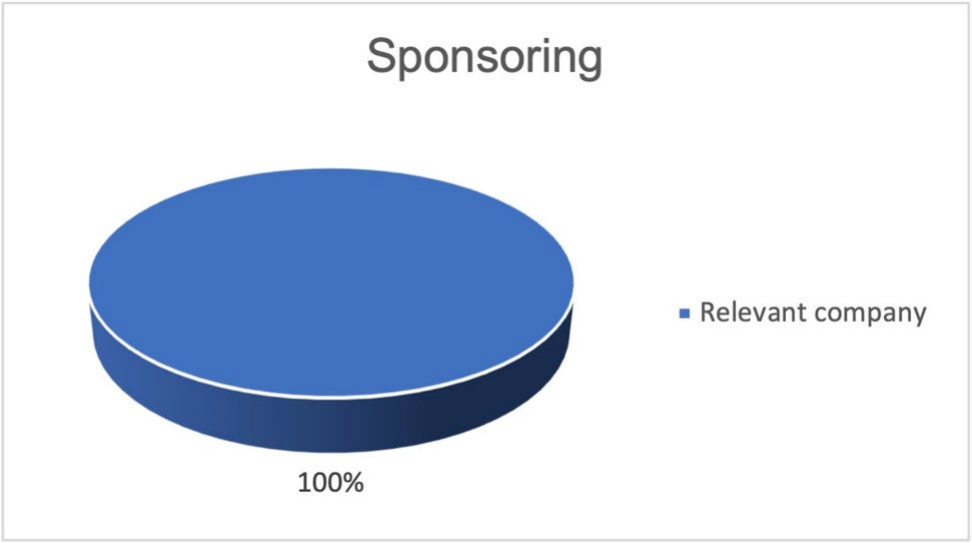
Proportion of industry-sponsored studies, in terms of all studies, that observed increased bioavailability of curcumin

Figure 14 shows that all publications related to the bioavailability of curcumin that successfully demonstrated their primary outcome measures were affected by industry sponsorship. Industrial sponsorship of studies with successfully proven primary outcome measures is significantly more frequent.

### Comparison of our study with three review articles

We compared our data with three review studies on this topic using a common set of parameters. All four studies are analyzed and compared in Table 3. One comprehensive study deals with the effect of curcumin on malignant diseases and the side effects of cancer chemotherapy and radiotherapy (Karaboğa Arslan et al. 2022). Twenty-one studies from the period 2010–2020 were analyzed. Sixteen of these studies addressed the effectiveness of curcumin in the treatment of malignant diseases and 5 studies addressed the effect of curcumin on the side effects caused by chemo and radiotherapy. It was concluded that curcumin shows a promising effect on malignant diseases and combating the side effects of radiation and chemotherapy. However, further long-term studies need to be conducted (Karaboğa Arslan et al. 2022). The analysis showed no significant efficacy of curcumin on malignant diseases in any study as well.

**Table 3:** Comparison between existing meta-analyses and our work

Study	Research question	Number of studies analyzed	Study selection procedure	Assessment of the quality of studies	Main results	Study bias	Publication date	Critical reference to bioavailability	References
The golden spice curcumin in cancer: A perspective on finalized clinical trials during the last 10 years	Analysis of the effect of turmeric on various types of cancer in studies in English from the period 2010 to 2020	21	Included were studies in English from PubMed, ScienceDirect, Google Scholar, and clinicaltrials.gov, in the period 2010–2020.	No critical study assessment procedure was applied	“The emerging data from clinical trials confirm that curcumin has considerable potential to treat cancer patients. However, it is not yet clear whether the long-term use of curcumin supplementation will show similar benefits”	No critical bias analysis procedure was applied	2022	Reference to the bioavailability of curcumin was made	Karaboğa Arslan et al. (2022)
Clinical effects of curcumin in enhancing cancer therapy: A systematic review	“This study aims to determine the Clinical effects of curcumin in different types of cancers using systematic review approach”	22	195 studies from the platforms SID, MagIran, IranMedex, IranDoc, Google Scholar, ScienceDirect, Scopus, PubMed and Web of Science (ISI) were filtered to 22 studies in a transparent screening process.	CONSORT checklist	“In a number of studies considered in this systematic review have shown that taking curcumin would increase the expression of anti-metastatic proteins”	CONSORT checklist	2020	No analysis of studies on bioavailability was performed	Mansouri et al. (2020)
A Systematic Review Assessing Clinical Utility of Curcumin with a Focus on Cancer Prevention	“To assess current knowledge on the broader potential for clinical efficacy of curcumin and in particular, in cancer prevention strategies”	314	Randomized clinical studies published in the English language, drug or placebo-controlled randomized trials including crossover trials; studies that used curcuminoids or turmeric preparations with specified content of curcumin or curcuminoids.	Analysis by means of methodological critique	“In the cancer prevention setting, there is evidence for positive clinical benefit despite overall participant numbers being low.”	Study biases were analyzed transparently	2021	No analysis of studies on bioavailability was performed	Howells et al. (2021)
Clinical trials on curcumin in relation to its bioavailability and effect on malignant diseases: a Critical analysis	“The aim of this study is to systematically review and critically analyze clinical studies related to the pharmacokinetics (or bioavailability) and to the use of curcumin in the treatment of malignant diseases”	52	Clinical trials listed on clinical trials.gov with reference to the bioavailability and effect of curcumin on malignant diseases.	Transparent analysis of the studies by applying different parameters of methodological critique	The analysis failed to convincingly demonstrate that curcumin has a significant, positive effect on the therapy of malignant diseases. Regarding to the increase of in bioavailability, positive results have been obtained, which are in proximity to the pharmaceutical industry. Independent studies could not achieve increased bioavailability of curcumin.	Study biases were analyzed transparently	2023	Critical analysis of the bioavailability in relation to the clinical efficacy of curcumin was performed	Khosravi and Seifert (2023, this paper)

Mansouri et al. (2020) published a systematic review on the effects of curcumin in tumor cells. A total of 22 studies were analyzed (Table 3). It was concluded that curcumin could be used as an effective combination in cancer therapy. The conclusions, different from ours, could be attributed to different data sources. In addition, we differentiated between the categories active treatment and treatment of concomitant complaints, which further narrowed down the positive results regarding the treatment of malignant diseases with curcumin. For example, the positive result of the review (Mansouri et al. 2020) regarding the improvement of skin problems in patients with malignant diseases would fall into the category treatment of concomitant complaints in our work. Accordingly, it would not be listed as a positive result of curcumin on malignant diseases. Furthermore, the different results could be due to differences in the analysis and resulting evaluation of the studies, in terms of method critique. In addition, in our work we have established a connection between the possible cancer therapy by curcumin and the simultaneous problem of bioavailability.

Howells et al. (2021) published a systematic review on 314 randomized clinical trials, specifically related to the prevention of malignant diseases through curcumin (Table 3). Of the analyzed studies, 100 identified significant changes within their participant groups regarding the primary outcome measures (Howells et al. 2021). Despite the substantial number of studies showing positive results, the authors concluded that factors such as the varying number of participants and the dosage of the supplement would prevent a more definitive conclusion (Howells et al. 2021). They found evidence for the use of curcumin in cancer prevention but also recommended further well-documented international multi-site studies (Howells et al. 2021).

Thus, the existing review papers on the topic of curcumin and malignant diseases yielded only partially convincing conclusions, at best. In our work, we have pursued the goal of illustrating a complete picture of the study situation on the clinical applicability of curcumin. This also includes the analysis of the bioavailability of curcumin, which has a major influence on the clinical efficacy of curcumin but is rarely in the focus of studies. But with the poor bioavailability of curcumin, systemic effects are hard to explain. Furthermore, we have evaluated the existing results on malignant diseases with the help of methodological critique and analysis of the study design (Fig. 3). We thus present a new interface between the bioavailability and the clinical effect of curcumin in relation to malignant diseases.

## Future studies and limitations of our study

Interest in curcumin is growing, and so is research (Fig. 3). Looking at all 293 studies listed on clinicaltrials.gov, the field of bioavailability plays a subordinate role at only 5.5% of all studies, which is in stark contrast to its relevance. The controversial studies on COX-2 and the in vitro findings on apoptosis in cell lines of T-cell lymphoma must be clarified by further research. The publications of the studies listed on clinicaltrials.gov are very rarely directly linked to each other and are therefore hard to find. We cannot guarantee to have found every publication through our research.

## Conclusions

Many of the studies listed on clinicaltrials.gov on the use of curcumin in malignant diseases did not yield any publications. Fifty percent of the publications on this topic did not investigate the actual active effect of curcumin on malignant diseases, but the effect of curcumin on the side effects of the therapy of malignant diseases (Fig. 7). Since 86% of the studies on the adverse effects deal with the already investigated anti-inflammatory effect of curcumin, no new research objective is being pursued here (Fig. 8). Rather, areas that have already been investigated were given a new context. Publications on active treatment of malignant disease by curcumin are strongly affected by study bias and rarely provide positive results (Table 1). Regardless of the indication, every in vivo study working with curcumin is burdened by the problem of low bioavailability. The publications examined that found an increase in bioavailability were 100% affected by industry sponsorship (Fig. 14). Furthermore, there are very few results that support each other. No study was identified showing that curcumin has a clinically relevant effect in tumor patients such as an increase in quality of life or even prolongation of life. Therefore, at the time being, there is no solid scientific basis for advertising the use of curcumin in tumor patients which is in stark contrast to the public hype about curcumin and the large number of scientific studies on this topic.

## Data Availability

All source data for this study are available upon reasonable request.

## Abbreviations

COX-2: Cyclooxygenase-2
NF-κB: Nuclear factor “kappa-light-chain-enhancer” of activated B-cells
MAG-OM3: Omega-3 fatty acids esterified in monoglycerides
PFS: Progression-free survival
